# A supervised topic embedding model and its application

**DOI:** 10.1371/journal.pone.0277104

**Published:** 2022-11-04

**Authors:** Weiran Xu, Koji Eguchi

**Affiliations:** Graduate School of Advanced Science and Engineering, Hiroshima University, Higashihiroshima, Hiroshima, Japan; Pontificia Universidad Catolica de Chile, CHILE

## Abstract

We propose rTopicVec, a supervised topic embedding model that predicts response variables associated with documents by analyzing the text data. Topic modeling leverages document-level word co-occurrence patterns to learn latent topics of each document. While word embedding is a promising text analysis technique in which words are mapped into a low-dimensional continuous semantic space by exploiting the local word co-occurrence patterns within a small context window. Recently developed topic embedding benefits from combining those two approaches by modeling latent topics in a word embedding space. Our proposed rTopicVec and its regularized variant incorporate regression into the topic embedding model to model each document and a numerical label paired with the document jointly. In addition, our models yield topics predictive of the response variables as well as predict response variables for unlabeled documents. We evaluated the effectiveness of our models through experiments on two regression tasks: predicting stock return rates using news articles provided by Thomson Reuters and predicting movie ratings using movie reviews. Results showed that the prediction performance of our models was more accurate in comparison to three baselines with a statistically significant difference.

## Introduction

Topic models are statistical machine learning models that find latent semantic structure in a corpus. They have been commonly applied as a tool for analyzing large amounts of text data in a variety of fields. Most topic models focus on the words that appear in documents. In some fields, however, a document is generally accompanied by a response, such as a movie review with a rating of the movie, or a financial news article accompanied by a financial indicator. Thus, to tackle such regression problems, we developed a supervised topic embedding model to infer latent topics predictive of the response.

Latent Dirichlet Allocation (LDA) [1] is a representative topic model which assumes a latent topic hidden behind each word in a document and infers the topics which compose the document by employing a hierarchical Bayesian structure. In LDA, the co-occurrence patterns of words can express semantic relevance when the corpus is large enough. However, the input documents in LDA are represented as bag-of-words (BoW), which have issues with high dimensionality and sparsity since the words are represented as one-hot representations that lack the notion of similarity between similar words. For instance, according to Dieng et al. [2], the quality of LDA topics decreases as the vocabulary size grows larger. Therefore, conventional topic models like LDA find latent topics from a corpus-level perspective with a drawback of igonoring word-level features because of the one-hot representations.

A solution to the drawback is word embedding, which introduces a semantic space where words are represented as *n*-dimensional vectors and the distance of the words can be used to measure the similarity of them, where *n* is much smaller than the vocabulary size. That is, word embedding maps words with similar meanings closely in a low-dimensional space where a vocabulary with tens of thousands is embedded. The semantic space is constructed by analyzing semantic similarity denoted by word co-occurrences in a sufficiently large corpus since words with similar contexts usually have similar meanings according to the distributional hypothesis [3].

Hence topic models and word embedding methods are both constructed by analyzing word co-occurrences but in different ways. A variety of approaches aiming to capture the global semantic structure and local word-level features by combining topic models and word embedding have been proposed in the literature [2, 4–8]. Such models have been shown to outperform LDA in terms of topic quality, predictive performance, and document classification tasks. In this paper, we extend TopicVec [5], a topic embedding model that seamlessly integrates topic models and word embedding, which is based on the idea that latent topics are also included in the word embedding space by combining the notion of topics with the generative word embedding model PSDVec [9]. The topic distributions for each document in TopicVec, like LDA, are assumed to be drawn from Dirichlet priors. In addition, each word in a document is assumed to be extracted from a link function that considers both surrounding context and global topics represented by word embeddings and topic embeddings respectively.

On the other hand, the topic distributions for each document suitably discovered by topic models can be leveraged for downstream tasks like regression problems. However, the topics learned by unsupervised topic models may be inappropriate to describe the coefficients of the post-processed regression since the supervisory signals such as numerical labels associated with documents are not involved in the topics learning procedure. Therefore, there exist approaches that share the goal of learning topics and regression coefficients jointly when each document is paired with a label or a response [10–13]. However, such models using sparse representations suffer from the same problem with LDA. By replacing the one-hot representations with dense vectors, the topics and the prediction accuracy could be improved intuitively.

This paper is extended from our preliminary work [14]. To the best of our knowledge, this study is the first to extend topic embedding models like TopicVec for regression tasks. Inspired by sLDA [10], in this paper, assuming a response variable is drawn from a Gaussian whose expectation is the inner product of the expectation of the topics for the corresponding document and the regression coefficients, we propose rTopicVec as a supervised topic embedding model for modeling the link between each document and a numerical label paired with it. Moreover, by assuming Gaussian priors to the regression coefficients, we propose a regularized version rTopicVec-Ridge. A variational Bayesian inference approach is used to simultaneously learn the parameters of both models, including the regression coefficients and latent variables. We conducted two experiments to verify the effectiveness of our proposed models. The objective of the first experiment was to predict stock return rates using news articles provided by Thomson Reuters and stock prices from the Tokyo Stock Exchange. The second experiment was to predict movie rating scores using movie reviews. In comparison to baseline models, our proposed models improved prediction performance significantly and also have the advantage of providing interpretability with latent topics for advanced regression analysis. The contributions of this work are summarized as follows:

1. We developed a supervised topic embedding model where words and topics are represented by embeddings and a regularized variant of the model. To the best of our knowledge, this work is the first to extend an unsupervised topic embedding model to a supervised one for regression tasks.
2. Our models learn topic parameters and regression parameters simultaneously and introduce word embeddings to improve the topic quality and prediction accuracy.
3. Our models outperformed three baseline models in prediction accuracy on two tasks that predict numerical labels associated with documents in two languages, respectively. The topic coherence was also improved by taking numerical labels into account.

## Related work

The model TopicVec [5] we extend in this paper is a topic embedding model incorporating a generative word embedding PSDVec [9] with latent topics. There exist other models that share the idea of combining topic models with word embedding methods. All of those are unsupervised models, while our model is a supervised extension of topic embedding.

### GaussianLDA

GaussianLDA proposed by Das et al. [4], uses pre-fitted word embeddings to benefit topic models. It replaces the categorical distributions representing topic-word distributions in LDA with multivariate Gaussian distributions so that topics and words share the same embedding space where the word embeddings are assumed to be drawn from a multivariate Gaussian centered at a topic embedding that is drawn from a multivariate Gaussian with zero mean and an inverse Wishart distribution as covariance.

### STE

Assuming that a word may have different representations under different topics, a unified framework STE (Skip-gram Topical word Embedding) proposed by Shi et al. [7] learns latent topics and topic-specific word embeddings jointly rather than learns them separately in a two-step way. The learned word embeddings are useful to address the issue of polysemy. They proposed two variants of STE by modeling each skip-gram in two different ways depending on whether the topics behind the two words in each skip-gram are the same or not.

### GPU-DMM

Focusing on analyzing short texts, Li et al. [6] proposed a topic model for shot texts that is based on Dirichlet Mixture Model with pre-fitted word embeddings incorporated by a generalized Pólya urn (GPU) model. The word embedding trained on a large corpus can supplement short text analysis where the context and word co-occurrence are limited. The GPU model promotes the semantically related words under the same topic, which is efficient for short text analysis.

### WEI-FTM

WEI-FTM is another topic model that uses pre-fitted word embeddings as prior knowledge to boost the topic quality when analyzing short texts proposed by Zhao et al. [8]. They assumed that the topic distributions over words are affected by the inner product of the word embeddings and topic embeddings. They also applied sparsity-enforcing prior on topics to make each of them focus on a subset of words rather than the whole vocabulary, leading to better topic quality.

### ETM

More recently, ETM (embedded topic model) developed by Dieng et al. [2] learns topics and the embeddings of them either fitting word embeddings jointly or using pre-fitted word embeddings by an amortized variational inference algorithm for which they replaced the Dirichlet with the logistic-normal distribution to model the topic distributions. They assumed that each word is generated according to the agreement between the word embeddings and the embedding of its assigned topic.

Regarding the supervised models that use topic representations of documents for regression problems, most of them mainly focus on extending LDA. Such models can be used to predict the label given an unlabeled document by inferring its latent topics. Mcauliffe et al. [10] proposed Supervised LDA (sLDA), in which the response paired with each document is presumed to be drawn from a Gaussian whose expectation is the product of the topic distribution of each document and the regression coefficients. MedLDA proposed by Zhu et al. [12] has a similar goal with sLDA and trains LDA with SVM by integrating the max-margin principle with the topic models. More recently, Wang et al. [13] proposed TAM, in which attention RNN is exploited to extend neural topic models for regression and classification tasks. While in our work, following sLDA, we propose rTopicVec that integrates the topic embedding model TopicVec with linear regression for regression problems and its regularized version rTopicVec-Ridge. We believe that our models should yield higher prediction accuracy than learning a regression model using the already estimated topic distributions as the explanatory variables, and also than sLDA, by involving word embeddings.

## Background

We briefly review the generative word embedding PSDVec [9] and topic embedding TopicVec [5] as the basic background of our work. The notations used in this paper are listed in Table 1.

**Table 1:** Notations.

Name	Description
***S***	Vocabulary{*s*_1_, ⋯, *s*_*W*_}
***V***	Embedding martix($v_{s_{1}},\cdots ,v_{s_{W}}$)
***D***	Document set{*d*_1_, ⋯, *d*_*M*_}
$v_{s_{m}}$	Embedding of word type *s*_*m*_
***μ***	Tiknov regularization coefficients (*μ*_1_, ⋯, *μ*_*W*_)
*h*_*mn*_	Bigram empirical probability of bigram (*s*_*m*_, *s*_*n*_)
$a_{s_{m}s_{n}},A$	Bigram residuals
***t***_*k*_, ***T***	Topic embeddings
*r*_*k*_, ***r***	Topic residuals
*w*_*ij*_	j-th word in document *d*_*i*_
*L*_*i*_	Length of document *d*_*i*_
*z*_*ij*_	Topic assignment of the *j*-th word in doc *d*_*i*_
***ϕ***_*i*_	Mixing proportions of topics in doc *d*_*i*_
***y***	response variables{*y*_1_, ⋯, *y*_*M*_}
***η***	regression coefficients{*η*_1_, ⋯, *η*_*K*_}

### PSDVec

Positive-Semidefinite Vectors (PSDVec) [9] is a generative word embedding method based on which TopicVec was developed. In PSDVec, the conditional distribution of a focus word given its context words is assumed to be factorized approximately into independent log-bilinear terms and it is defined by the following link function:

$$\begin{array}{c} P(w_{ij}|w_{i,j-c}:w_{i,j-1})\approx P(w_{ij})\;\text{exp}\;\{v_{w_{ij}}^{⊤}\sum_{l=j-c}^{j-1}v_{w_{il}}+\sum_{l=j-c}^{j-1}a_{w_{ij}w_{il}}\}. \end{array}$$ (1)

The link function connects the word embeddings with the corpus statistics. Here, the focus word *w*_*ij*_ is assumed to be generated depending on context of size *c*. $v_{w_{ij}}^{T}v_{w_{il}}$ captures the linear correlations of two words and bigram residual $a_{w_{ij}w_{il}}$ captures the non-linear part.

Given the hyperparameter ***μ*** = (*μ*_1_, ⋯, *μ*_*W*_) and a weight function on the bigram probability *f*(*h*_*mn*_), the generative process for the corpus is as follows:

1. For each word type *s*_*m*_, draw the embedding $v_{s_{m}}$ from $N(0,\frac{1}{2\mu_{m}}I)$;
2. For each bigram (*s*_*m*_, *s*_*n*_), draw $a_{s_{m}s_{n}}$ from $N(0,\frac{1}{2f(h_{mn})})$;
3. For each document *d*_*i*_, draw the *j*-th word *w*_*ij*_ from vocabulary ***S*** according to the probability defined by (1).

We omit the derivation process here. The derived optimization objective is to fit pointwise mutual information $\text{PMI}\left(s_{m},s_{n}\right)=\text{log}\;\frac{P(s_{m},s_{n})}{P\left(s_{m}\right)P\left(s_{n}\right)}$ using $v_{s_{n}}^{⊤}v_{s_{m}}$, and it is optimized by a block coordinate descent algorithm.

### TopicVec

TopicVec [5] was developed by taking topics into account in PSDVec described in the previous section. The conditional distribution of the focus word in TopicVec is therefore affected by its context as well as the topic assigned to the word and it is defined by the following function:

$$\begin{array}{cc} P(w_{ij}|w_{i,j-c}:w_{i,j-1},z_{ij},d_{i})\approx & P(w_{ij})\text{exp}\{v_{w_{ij}}^{⊤}(\sum_{l=j-c}^{j-1}v_{w_{il}}+t_{z_{ij}})\\ & +\sum_{l=j-c}^{j-1}a_{w_{il}w_{ij}}+r_{z_{ij}}\}. \end{array}$$ (2)

Here, $t_{z_{ij}}$ is the embedding of the topic assigned to the focus word and can be treated as one of the context words. $r_{z_{ij}}$ is the residual of the topic *z*_*ij*_. With the link function, the distance between each word and each topic encodes the relevance of them in the embedding space. The generative process of TopicVec is as follows:

1. For each topic *k*, randomly draw a topic embedding **t**_*k*_, each element of which is sampled from the standard Gaussian N(0,1).
2. For each document *d*_*i*_:
  (a) Draw the mixing proportions ***ϕ***_*i*_ from the Dirichlet prior Dir(***α***);
  (b) For the *j*-th word:
    i Draw topic assignment *z*_*ij*_ from the categorical distribution Cat(***ϕ***_*i*_);
    ii Draw word *w*_*ij*_ from vocabulary *S* according to *P*(*w*_*ij*_|*w*_*i*,*j*−*c*_: *w*_*i*,*j*−1_, *z*_*ij*_, *d*_*i*_).

**Fig 1:**
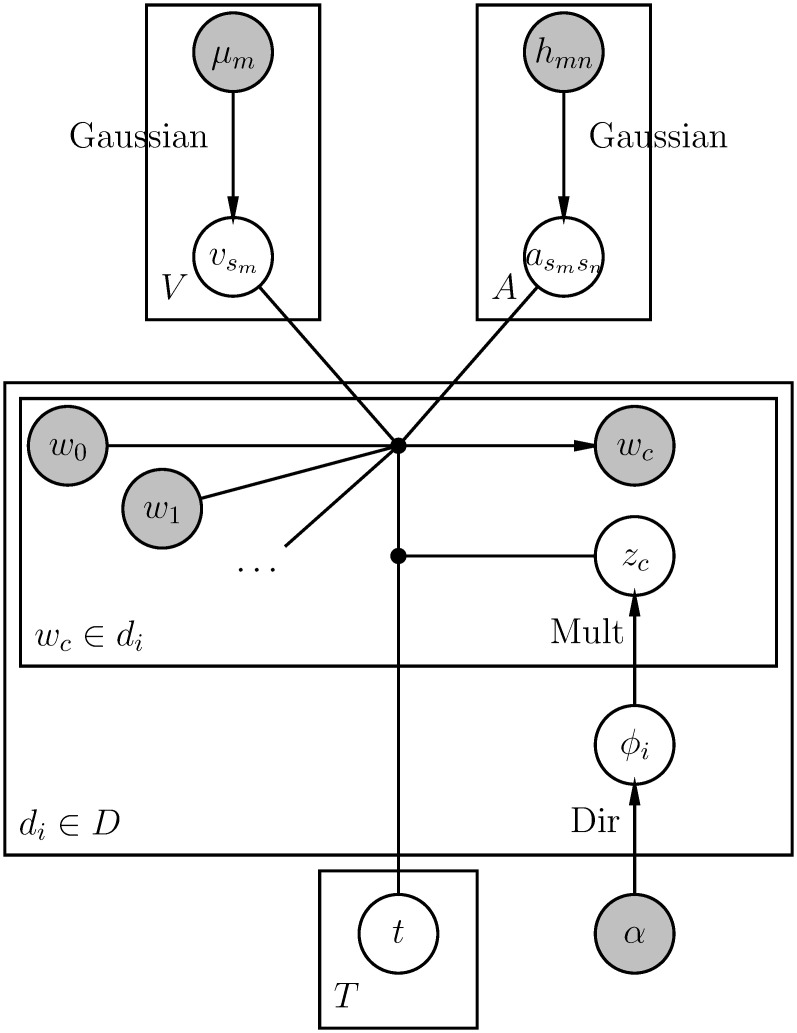
Graphical model of TopicVec.

Fig 1 presents a graphical model for the generative process above.

The complete data loglikelihood of the whole corpus (the full joint log-probability of the corpus ***D***, word embeddings ***V***, bigram residuals ***A***, topic embeddings ***T***, topic assignments ***Z***, and topic distributions ***ϕ***) can be written as

$$\begin{array}{cc} & \text{log}\;p(D,A,V,Z,T,\phi |\alpha ,\gamma ,\mu )\\ = & C_{0}-\text{log}\;Z\left(H,\mu \right)-{\left\|A\right\|}_{f(H)}^{2}-\sum_{m=1}^{W}\mu_{m}\|v_{s_{m}}{\|}^{2}\\ & +\sum_{i=1}^{M}\{\sum_{k=1}^{K}\text{log}\;\phi_{ik}(b_{ik}+\alpha_{k}-1)+\sum_{j=1}^{L_{i}}(r_{z_{ij}}\\ & +v_{w_{ij}}^{⊤}(\sum_{l=j-c}^{j-1}v_{w_{il}}+t_{z_{ij}})+\sum_{l=j-c}^{j-1}a_{w_{il}w_{ij}})\}, \end{array}$$ (3)

where $b_{ik}=\sum_{j=1}^{L_{i}}\delta \left(z_{ij}=k\right)$ indicates the number of words assigned to topic *k*. *C*_0_ is constant given the hyperparameters.

Given the hyperparameters ***α***, *γ*, and ***μ***, the optimal ***V***, ***T***, and *p*(***Z***, ***ϕ***|***D***, ***A***, ***V***, ***T***) are estimated to maximize the loglikelihood as follows:

**Step1 *V*** and ***A*** are optimized using the original PSDVec;
**Step2** Given optimal ***V*** and ***A***, the optimal ***T*** and *p*(***Z***, ***ϕ***|***D***, ***A***, ***V***, ***T***) are optimized using the loglikelihood function.

Since the posterior *p*(***Z***, ***ϕ***|***D***, ***T***) is analytically intractable, the posterior is approximated by the variational distribution *q*(***Z***, ***ϕ***; ***π***, ***θ***) = *q*(***ϕ***; ***θ***)*q*(***Z***; ***π***). Here, the KL divergence is introduced and the estimation task is replaced with the problem of maximizing the variational lower bound L(q,T):

$$\begin{array}{cc} \text{KL}(q\|p)= & \text{log}\;p\left(D|T\right)-(E_{q}\left[\text{log}\;p\left(D,Z,\phi |T\right)\right]+H\left(q\right))\\ = & \text{log}\;p(D|T)-L(q,T) \end{array}$$ (4)

where H(q) is the entropy of *q*. The variational lower bound L(q,T) is as follows:

$$\begin{array}{cc} L(q,T)= & \sum_{i=1}^{M}\{\sum_{k=1}^{K}(\sum_{j=1}^{K}\pi_{ij}^{k}+\alpha_{k}-1)(\psi (\theta_{ik})-\psi (\theta_{i0}))\\ & +\text{Tr}(T^{⊤}\sum_{j=1}^{L_{i}}v_{w_{ij}}\pi_{ij}^{⊤})+r^{⊤}\sum_{j=1}^{L_{i}}\pi_{ij}\}+H\left(q\right)+C_{1}. \end{array}$$ (5)

Here, $C_{1}=C_{0}-\text{log}\;Z\left(H,\mu \right)-{\left\|A\right\|}_{f(H)}^{2}-\sum_{m=1}^{W}\mu_{m}\|v_{s_{m}}{\|}^{2}+\sum_{i,j=1}^{M,L_{i}}\left(v_{w_{ij}}^{⊤}\sum_{l=j-c}^{j-1}v_{w_{il}}+\sum_{l=j-c}^{j-1}a_{w_{il}w_{ij}}\right)$ is constant. Then the generalized EM algorithm is used to find the optimal q* and T* that maximize L(q,T) as shown in Algorithm 1. Here, ***u*** is the unigram probability of the words occurring in the corpus. $\lambda \left(\ell ,\sum_{i=1}^{M}L_{i}\right)=\frac{L_{0}\lambda_{0}}{\ell \cdot \text{max}\{\sum_{i=1}^{M}L_{i},L_{0}\}}$ is the learning rate, where *ℓ* is the number of iterations in the learning process, *L*_0_ is a predetermined threshold of the number of words, and λ_0_ is the initial value of λ.

**Algorithm 1** The generalized EM algorithm

**Initialize *T***, ***r***, ***θ***

**repeat**

 **E-Step**:

$\pi_{ij}^{k}\propto \text{exp}\{\psi (\theta_{ik})+v_{w_{ij}}^{⊤}t_{k}+r_{k}\}$

$\theta_{ik}=\sum_{j=1}^{L_{i}}\pi_{ij}^{k}+\alpha_{k}$

 **M-Step**:

$T_{\text{new}}=T+\lambda (l,\sum_{i=1}^{M}L_{i})\frac{\partial L(q,T)}{\partial T}$

  ***r*** = −log(***u*** exp{***V***^⊤^***T***})

**until** converged

## Supervised topic embedding model

In this section, we introduce our supervised topic embedding model for regression, rTopicVec, which incorporates regression into TopicVec mentioned in the previous section, and its regularized version, rTopicVec-Ridge.

### Generative process

In rTopicVec, we assume that the document *d*_*i*_ and an accompanying response variable *y*_*i*_ are generated following the generative process as follows:

1. Generate words and topic assignments of each document *d*_*i*_ following the generative process of TopicVec;
2. Draw response variable $y_{i}∼N\left(\eta^{⊤}\bar{Z_{i}},\delta^{2}\right)$.

**Fig 2:**
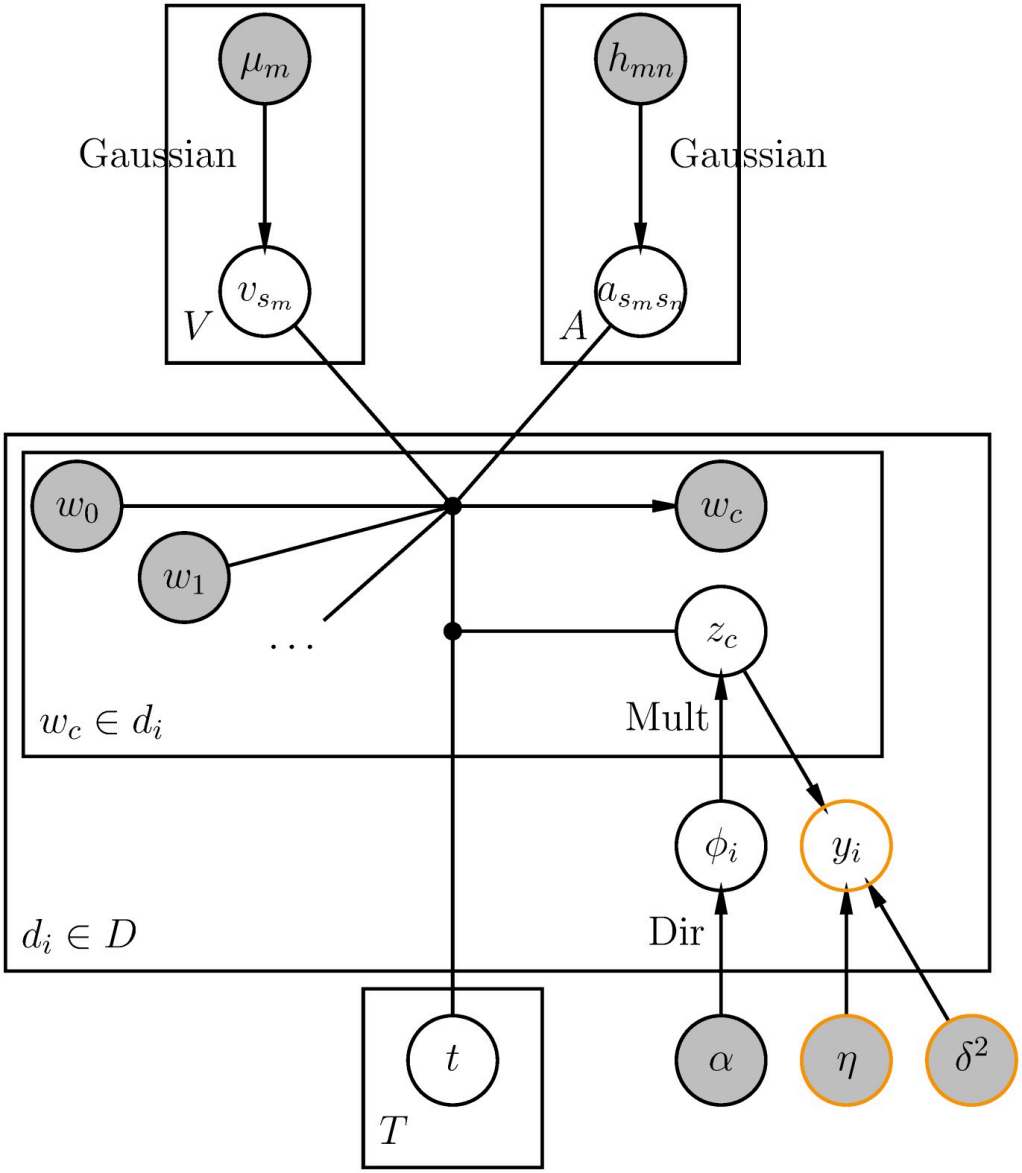
Graphical model of rTopicVec.

Step 2 is newly added here to generate a response variable given the latent topics of the document generated in step 1. The expectation of the Gaussian distribution in step 2 is the inner product of the regression coefficients ***η*** and the expectation of topic assignments $\bar{Z_{i}}$ for *d*_*i*_. Fig 2 presents a graphical model of rTopicVec. The orange colored circles correspond to step 2 in the generative process, indicating the nodes added to the previous graphical model in Fig 1. To prevent overfitting, we propose a regularized version named rTopicVec-Ridge, by further assuming standard normal priors on the coefficients ***η***, which is equivalent to ridge regression.

### Estimation of parameters

For rTopicVec, we estimate the parameters including the ones for regression using the generalized EM algorithm after deriving the loglikelihood function similar to TopicVec.

First, we rewrite the complete data loglikelihood in (3) to include response variables ***y*** = {*y*_*i*_} as:

$$\begin{array}{cc} & \text{log}\;p(D,A,V,Z,T,\phi ,y|\alpha ,\gamma ,\mu ,\eta ,\delta^{2})\\ & =C_{0}-\text{log}\;Z\left(H,\mu \right)-{\left\|A\right\|}_{f(H)}^{2}-\sum_{m=1}^{W}\mu_{m}\|v_{s_{m}}{\|}^{2}\\ & +\sum_{i=1}^{M}\{\sum_{k=1}^{K}\text{log}\;\phi_{ik}(b_{ik}+\alpha_{k}-1)-\frac{1}{2}\text{log}\;\left(2\pi \delta^{2}\right)\\ & -\frac{1}{2\delta^{2}}\left(y_{i}^{2}-2y_{i}\eta^{⊤}{\bar{Z}}_{i}+\eta^{⊤}{\bar{Z}}_{i}{\bar{Z}}_{i}^{⊤}\eta \right)\\ & +\sum_{j=1}^{L_{i}}(v_{w_{ij}}^{⊤}(\sum_{l=j-c}^{j-1}v_{w_{il}}+t_{z_{ij}})+\sum_{l=j-c}^{j-1}a_{w_{il}w_{ij}}+r_{z_{ij}})\}. \end{array}$$ (6)

Then by introducing a variational distribution *q*(***Z***, ***ϕ***; ***π***, ***θ***) = *q*(***ϕ***; ***θ***)*q*(***Z***; ***π***) as in TopicVec, the expectation of the variational distribution of the loglikelihood of the response variable *y*_*i*_ is obtained by

$$\begin{array}{cc} E_{q}[\text{log}\;p(y_{i}|Z_{i},\eta ,\delta^{2})]= & -\frac{1}{2}\text{log}\;(2\pi \delta^{2})\\ & -\frac{1}{2\delta^{2}}(y_{i}^{2}-2y_{i}\eta^{⊤}E_{q}\left[{\bar{Z}}_{i}\right]+\eta^{⊤}E_{q}[{\bar{Z}}_{i}{\bar{Z}}_{i}^{⊤}]\eta ), \end{array}$$ (7)

where

$$\begin{array}{c} E_{q}\left[{\bar{Z}}_{i}\right]={\bar{\pi }}_{i}=\frac{1}{L_{i}}\sum_{j=1}^{L_{i}}\pi_{ij}, \end{array}$$

$$\begin{array}{cc} & E_{q}[{\bar{Z}}_{i}{\bar{Z}}_{i}^{⊤}]=\frac{1}{{L_{i}}^{2}}(\sum_{j=1}^{L_{i}}\sum_{j^{{}^{\prime }}\neq j}\pi_{ij}\pi_{ij^{{}^{\prime }}}^{⊤}+\sum_{j=1}^{L_{i}}\text{diag}\{\pi_{ij}\}). \end{array}$$

Thus, the objective $L_{\text{r}}\left(q,T\right)$ is obtained by adding (7) to (5):

$$\begin{array}{cc} L_{\text{r}}\left(q,T\right)= & \sum_{i=1}^{M}\{\sum_{k=1}^{K}(\sum_{j=1}^{L_{i}}\pi_{ij}^{k}+\alpha_{k}-1)(\psi (\theta_{ik})-\psi (\theta_{i0}))\\ & +(-\frac{1}{2}\text{log}\;\left(2\pi \delta^{2}\right)-\frac{y_{i}^{2}}{2\delta^{2}})+\text{Tr}(T^{⊤}\sum_{j=1}^{L_{i}}v_{w_{ij}}\pi_{ij}^{⊤})\\ & +(r^{⊤}+\frac{y_{i}\eta^{⊤}}{L_{i}\delta^{2}})\sum_{j=1}^{L_{i}}\pi_{ij}\\ & +(-\eta^{⊤}\cdot \frac{1}{2{L_{i}}^{2}\delta^{2}}(\sum_{j=1}^{L_{i}}\sum_{j^{{}^{\prime }}\neq j}^{L_{i}}\pi_{ij}\pi_{ij^{{}^{\prime }}}^{⊤}+\sum_{j=1}^{L_{i}}\text{diag}\left\{\pi_{ij}\right\})\eta )\}\\ & +H\left(q\right)+C_{1} \end{array}$$ (8)

Here, *ψ*(⋅) is the digamma function. *θ*_*ik*_ and ***T*** are updated following the corresponding equations in E-step and M-step in Algorithm 1 respectively.

The solution is obtained by setting the partial derivative w.r.t. $\pi_{ij}^{k}$ to 0 after isolating the terms containing $\pi_{ij}^{k}$:

$$\begin{array}{c} \pi_{ij}^{k}\propto \text{exp}\{\psi \left(\theta_{ik}\right)+v_{w_{ij}}^{⊤}t_{k}+r_{k}+\frac{y_{i}\eta_{k}}{L_{i}\delta^{2}}-\frac{2\eta^{⊤}\Pi_{i,-j}^{\left(k\right)}\eta +{\left(\eta_{k}\right)}^{2}}{2{L_{i}}^{2}\delta^{2}}\}, \end{array}$$ (9)

where

$$\begin{array}{cc} \Pi_{i,-j}^{\left(k\right)}≔ & \sum_{j^{{}^{\prime }}\neq j}^{L_{i}}\pi_{ij^{{}^{\prime }}}(0^{\left(1\right)},\cdots ,1^{\left(k\right)},\cdots ,0^{\left(K\right)}{)}^{⊤}\\ + & (0^{\left(1\right)},\cdots ,1^{\left(k\right)},\cdots ,0^{\left(K\right)})\sum_{j^{{}^{\prime }}\neq j}^{L_{i}}\pi_{ij^{{}^{\prime }}}^{⊤} \end{array}$$

is the partial derivative of $\sum_{j=1}^{L_{i}}\sum_{j^{{}^{\prime }}\neq j}^{L_{i}}\pi_{ij}\pi_{ij^{{}^{\prime }}}^{⊤}$ w.r.t. $\pi_{ij}^{k}$.

Eq (7) contains the regression parameters in the learning objective. To involve the learning of the bias term in regression, we define a *M* × (*K* + 1) matrix *A* whose row is a topic proportion vector of a document attached by a 1: $\left({\bar{Z}}_{i},1\right)$ with the (*K* + 1)-th element corresponding to the bias. Over the whole corpus, Eq (7) can be rewritten as

$$\begin{array}{cc} & \eta^{{}^{\prime }}=\text{Concat}\left(\eta ,\eta_{\text{bias}}\right),\\ & E_{q}\left[\text{log}\;p\left(y|A,\eta^{{}^{\prime }},\delta^{2}\right)\right]=-\frac{M}{2}\text{log}\;\left(2\pi \delta^{2}\right)-\frac{1}{2\delta^{2}}E_{q}[{\left(y-A\eta^{{}^{\prime }}\right)}^{⊤}\left(y-A\eta^{{}^{\prime }}\right)], \end{array}$$ (10)

where ***η***′ is obtained by the function Concat(⋅) that concatenates the bias term to the end of the coefficients voctor ***η***. Taking the partial derivative w.r.t. ***η***′ and *δ*^2^ and setting them to 0, we obtain the following to update ***η***′ and *δ*^2^:

$$\begin{array}{c} \eta_{\text{new}}^{{}^{\prime }}=(E_{q}[A^{⊤}A]{)}^{-1}E_{q}[A{]}^{⊤}y, \end{array}$$ (11)

$$\begin{array}{c} \delta_{\text{new}\;}^{2}=\frac{1}{M}\{y^{⊤}y-y^{⊤}E_{q}\left[A\right](E_{q}[A^{⊤}A]{)}^{-1}E_{q}{\left[A\right]}^{⊤}y\} \end{array}$$ (12)

where we define $\pi_{ij}^{{}^{\prime }}≔\text{Concat}\left(\pi_{ij},1\right)$ to involve the bias term, and correspondingly

$$\begin{array}{c} E[A]=\frac{1}{L_{i}}\sum_{j=1}^{L_{i}}\pi_{ij}^{{}^{\prime }}, \end{array}$$

$$\begin{array}{cc} & E[A^{⊤}A]=\sum_{i=1}^{M}(\frac{1}{{L_{i}}^{2}}(\sum_{j=1}^{L_{i}}\sum_{j^{{}^{\prime }}\neq j}^{L_{i}}\pi_{ij}^{{}^{\prime }}\pi_{ij^{{}^{\prime }}}^{{}^{\prime }⊤}+\sum_{j=1}^{L_{i}}\text{diag}\left\{\pi_{ij}^{{}^{\prime }}\right\})). \end{array}$$

For rTopicVec-Ridge, we use MAP estimation by adding ℓ_2_ regularization to Eq (10). Then similarly we obtain the following:

$$\begin{array}{c} \eta_{\text{new}}^{{}^{\prime }}=(E_{q}[A^{⊤}A]+\lambda {\text{I}}^{\text{mod}}{)}^{-1}E_{q}[A{]}^{⊤}y, \end{array}$$ (13)

$$\begin{array}{c} \delta_{\text{new}}^{2}=\frac{1}{M}\{y^{⊤}y-y^{⊤}E_{q}\left[A\right](E_{q}[A^{⊤}A]+\lambda {\text{I}}^{\text{mod}}{)}^{-1}E_{q}{\left[A\right]}^{⊤}y\} \end{array}$$ (14)

where λ is the strength of the regularizer, and **I**^mod^ = diag(1^(1)^, ⋯, 1^(*K*)^, 0^(*K*+1)^) implies that the bias corresponding to the last element is excluded from the regularization.

## Experimental results

To evaluate the prediction performance of our proposed models, we performed experiments on two prediction problems. The first is to predict stock return rates using news articles provided by Thomson Reuters and stock prices from the Tokyo Stock Exchange. The second experiment, as performed by Mcauliffe et al. [10], is to predict movie rating scores using movie reviews. For each experiment, we first determined the number of topics for optimal prediction performance through validation tests with our proposed model rTopicVec.

We compared the performance of the proposed models rTopicVec and rTopicVec-Ridge with the following three baseline models:

**TopicVec+LR**: Perform linear regression as post-process using topics learned by TopicVec [5] as a baseline.
**TopicVec+Ridge**: Perform ridge regression as post-process using topics learned by TopicVec as a baseline.
**sLDA**: Supervised topic model [10] using BoW representations as a baseline.

The word embeddings ***V*** for the two TopicVec-based models in the two experiments were trained by PSDVec using Japanese Wikipedia and English Wikipedia following PSDVec [9]. The Dirichlet hyperparameter ***α*** is fixed to (0.1, ⋯, 0.1) [15] for all models in both experiments. The regularizer λ is set to 1 for rTopicVec-Ridge and TopicVec+Ridge.

### Stock price return rates prediction

#### Setup

For text data, we used financial articles in Japanese distributed by Thomson Reuters from January 2015 to June 2017. We preprocessed the corpus by removing intractable tables and unneeded expressions, and performing morphological analysis using MeCab with mecab-ipadic-NEologd [16–18], a dictionary of neologisms and named entities, to segment words. We replaced stop words such as particles and conjunctions with * as the link function learns conditional distribution of a word in a context window. Moreover, we excluded low-frequency words occurring in fewer than five documents and documents of shorter than 50 words.

As response variables associated with the financial articles, the stock return rate of the company mentioned in each article is defined as following using the Tokyo Stock Exchange’s historical data for stock prices:

$$\begin{array}{cc} R & =\frac{V_{f}-V_{f}^{{}^{\prime }}}{V_{f}^{{}^{\prime }}}, \end{array}$$

where *V*_*f*_ is the final value on the day **after** the article was published and $V_{f}^{{}^{\prime }}$ is the final value on the day **before** the article was published. When multiple companies appeared in one article, we sorted the return rates of those companies in descending order, then removed those whose absolute value was lower than the mean plus one standard deviation since the return rates of such companies may have not been affected by the content of the article. Articles that mentioned more than five companies were excluded because they likely focused on industry trends rather than specific companies.

As shown in Table 2, we divided the articles into five collections: those from the first half of 2015 (H1 2015), the latter half of 2015 (H2 2015), the first half of 2016 (H1 2016), the latter half of 2016 (H2 2016), and the first half of 2017 (H1 2017). Then we further divided each of the collections into two-monthly segments and prepared four preprocessed datasets in time order. We assumed that the topics in the financial markets would change gradually over time. Therefore, we set the data for adjacent terms to overlap by one month to capture this nature. We also show in the table the number of documents contained in each term of each dataset, as well as the average number of words per document (some of the documents were short as the * aforementioned were not included. Nevertheless, there is no unfairness in the comparison of the models.).

**Table 2:** Overview of datasets for stock price prediction.

	Training Sets (80% for training, 20% for validation)			Test Sets (Open Test)
Term	1	2	3	4
Dataset1 (H1 2015)	Jan.∼Feb.	Feb.∼Mar.	Mar.∼Apr.	May∼June
# of documents	982	1009	1060	856
Avg. # of words/document	63	73	79	82
Dataset2 (H2 2015)	Jul.∼Aug.	Aug.∼Sep.	Sep.∼Oct.	Nov.∼Dec.
# of documents	857	667	738	656
Avg. # of words/document	70	66	64	62
Dataset3 (H1 2016)	Jan.∼Feb.	Feb.∼Mar.	Mar.∼Apr.	May∼June
# of documents	633	698	663	599
Avg. # of words/document	67	65	64	68
Dataset4 (H2 2016)	Jul.∼Aug.	Aug.∼Sep.	Sep.∼Oct.	Nov.∼Dec.
# of documents	636	515	590	589
Avg. # of words/document	75	69	75	81
Dataset5 (H1 2017)	Jan.∼Feb.	Feb.∼Mar.	Mar.∼Apr.	May∼June
# of documents	720	728	726	673
Avg. # of words/document	77	68	71	68

To determine the optimal number of topics to be used in the open tests, 20% of the data in each training set were randomly drawn as hold-out for validation with ten different numbers of topics *K* ∈ {5, 10, 15, 20, 25, 30, 35, 40, 45, 50}. A validation test on the data of an overlapped month was performed with the model parameters learned by the latter of two datasets containing the data of this month, e.g., the validation test on Feb. 2016 data was performed with the model parameters learned in Term 2 rather than those learned in Term 1.

The model parameters that would be used in the open tests on Term 4 would be learned with 100% of the data in each training set and the optimal number of topics K* determined by the validation tests for rTopicVec. During the training procedure, for Term 1, topic embeddings ***T*** were initialized randomly from the standard Gaussian distribution, and the variational parameter ***π*** was randomly initialized following a Dirichlet distribution. For Term 2, ***T*** were initialized by the ones learned on Term 1. We followed the same procedure for the following terms, but we used ***T*** and ***η*** estimated with the previous term as the initial states of ***T*** and ***η***, respectively. The regression coefficient ***η*** was updated every five iterations, and we used the same experimental procedure for the other models as that for rTopicVec.

For models except sLDA in the open tests, with the optimal K*, we used TopicVec to estimate the topics on Term 4 with ***T*** learned from Term 3 as the initial ***T***. While for sLDA in the open tests, with the optimal K*, we used LDA to learn the topics on Term 4 with the topic-terms distributions ***β*** learned from Term 3 as the initial ***β***.

For all experiments, the convergence condition was that the rate of change of ***π*** (or ***ϕ*** in sLDA) must be lower than 0.1% three times in a row during learning.

The response variables are predicted as following:

$$\begin{array}{c} \pi_{ij}^{\prime }=\text{Concat}\left(\pi_{ij},1\right) \end{array}$$

$$\begin{array}{c} {\hat{y}}_{i}=\eta^{⊤}E_{q}\left[{\bar{Z}}_{i}\right]+\eta_{\text{bias}}=\eta^{{}^{\prime }⊤}\frac{1}{L_{i}}\sum_{j=1}^{L_{i}}\pi_{ij}^{\prime } \end{array}$$

To measure the performance, we used the mean squared error (MSE) between the predicted response variables and the ground truth:

$$\begin{array}{c} \text{MSE}=\frac{1}{M}\sum_{i=1}^{M}{\left(y_{i}-{\hat{y}}_{i}\right)}^{2} \end{array}$$

#### Results

**Fig 3:**
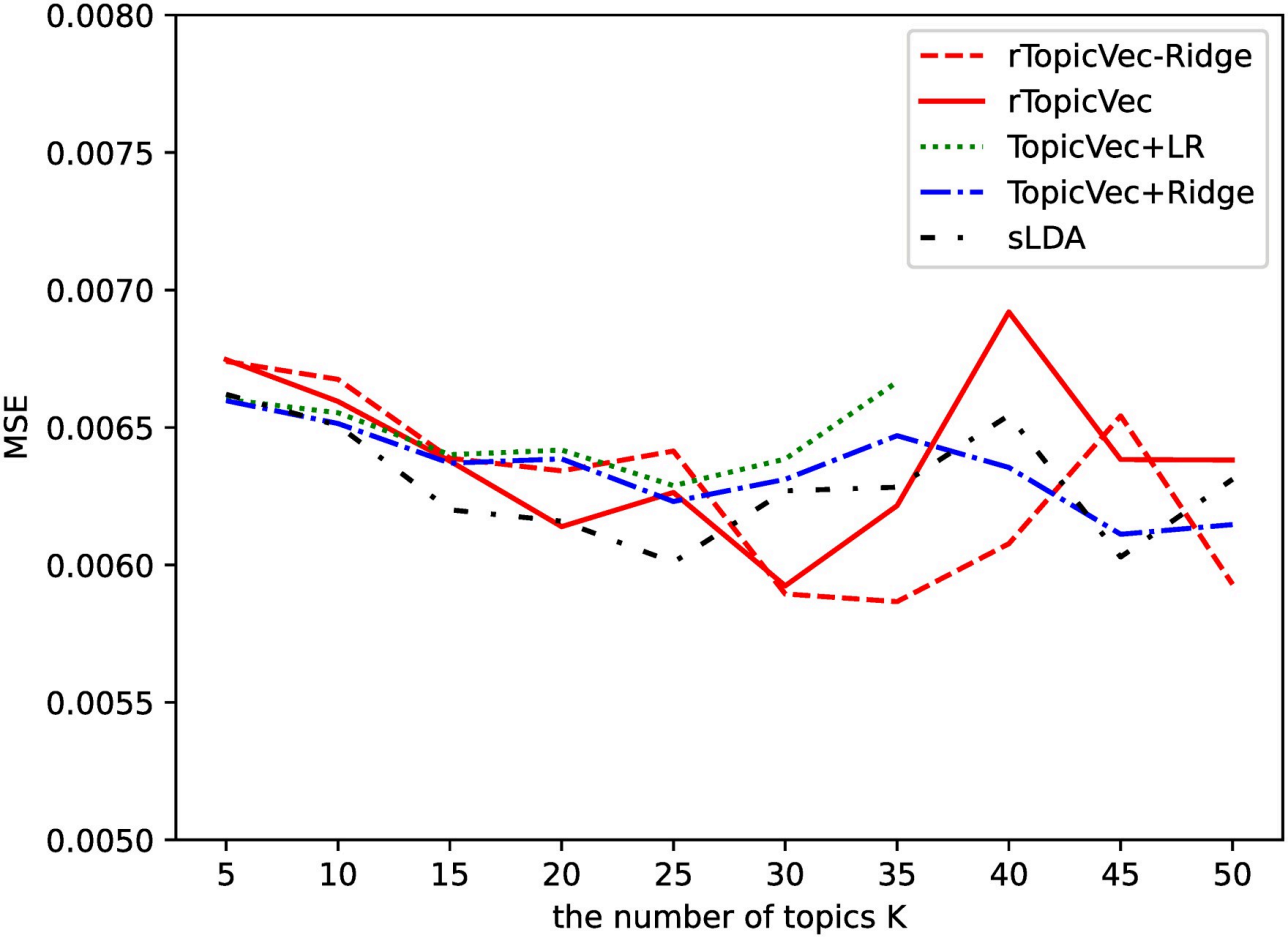
Average MSE for varying number of topics *K* on validation sets of stock return rates prediction.

Fig 3 shows the MSEs obtained by the five models as the average result of the five validation tests. The solid line which denotes rTopicVec shows that the proposed model yields higher prediction accuracy when *K* = 30 on average for the five validation tests. The results of TopicVec+LR when *K* > 35 are not shown here since the coefficients are too large due to overfitting. Besides, the two models whose regression coefficients are penalized have higher prediction accuracy when *K* is larger than 30.

Table 3 presents the topics with two of the highest absolute values of the regression coefficients learned by all the models on Term 3 in Dataset 2 when *K* = 30. Each of the topics is represented by the ten most relevant words translated from the original Japanese. The topics of TopicVec+LR are not shown here because overfitting during the linear regression learning leads to coefficients that are too large. We argue that the topics learned by our proposed models presented here are more coherent and reflect a rise/drop on stock price return rates.

**Table 3:** Top 10 words of two topics with highest absolute values of regression coefficients.

Model	Top 10 words
rTopicVec	coefficient: 0.092
	hot, stock, material, favor, rebound, leading, buying, the company, dividend, announcement
	coefficient: -0.055
	disappoint, downward revision, hot, stock, linking, sound, deficit, fluctuation, bring down, reverse
rTopicVec-Ridge	coefficient: 0.062
	favor, linking, increased profit, forecast, contribute, raise, hot, stock, dividend, achievement
	coefficient: -0.041
	disappoint, hot, stock, rebound, softness, reverse, sound, newspaper, report, report
TopicVec+Ridge	coefficient: 0.104
	acquisition, treasury stock, stock, self, stock, conduct, issue, total, upper limit, hold
	coefficient:-0.071
	money, rebuild, procurement, sponsor, debt, insolvency, investment, fund, cost, group
sLDA	coefficient: 0.078
	dollar, near, the U.S.A., euro, not, rise, domestic, increase in interest rates, financial institutions, the day before
	coefficient: -0.063
	announcement, forecast, linking, stock, hot, downward revision, disappoint, deficit, forecast, revision

These words are translated from the original Japanese into English.

Since K* = 30 was the optimal number of topics in validation tests, we performed open tests on test sets when K* = 30. Table 4 shows that in predicting the return rates of the unlabeled articles, one or both of the proposed models are more accurate than the baselines in four out of the five open tests. We also performed the Wilcoxon signed-rank test and the Paired-t test between the predicted response variables of our proposed models and those of the three baseline models. We found that four out of the five cases in which at least one of our proposed models had lower MSE values, and the p-values were less than 10%, indicating that the prediction performance of our models was more accurate than the three baseline models with a statistically significant difference. However, the MSE of rTopicVec was marginally higher than that of the baselines on Term 4 of Dataset 5, which may be due to the slight overfitting that occurred during the training process, while was mitigated by *ℓ*_2_ regularization shown by rTopicVec-Ridge. Thus we argue that the proposed models offers the advantage of explainability on the relationship between the latent topics and regression coefficients.

**Table 4:** MSE and sample standard deviation on the three test sets of stock price prediction when *K* = 30.

	May∼June 2015	Nov.∼Dec. 2015	May∼June 2016	Nov.∼Dec. 2016	May∼June 2017
TopicVec+LR	$0.00540_{\pm 0.03061}^{†,‡}$	−^‡^	$0.00857_{\pm 0.04040}^{†}$	−^†^	$0.01350_{\pm 0.07111}^{‡}$
TopicVec+Ridge	$0.00523_{\pm 0.03088}^{†,‡}$	$0.00495_{\pm 0.01283}^{‡}$	0.00845_±0.04107_	$0.00856_{\pm 0.02451}^{†}$	$0.01346_{\pm 0.07102}^{‡}$
sLDA	$0.00562_{\pm 0.03090}^{†}$	$0.00484_{\pm 0.01395}^{†}$	**0.00776**_±0.03823_	**0.00818**_±0.02457_	$0.01367_{\pm 0.07353}^{‡}$
*rTopicVec*	**0.00493**_±0.03100_	**0.00474**_±0.01252_	0.00854_±0.04138_	0.00839_±0.02509_	0.01369_±0.07296_
*rTopicVec-Ridge*	**0.00498**_±0.03193_	**0.00436**_±0.01159_	0.00800_±0.04101_	**0.00818**_±0.02419_	**0.01340**_±0.07097_

Bold face indicates the best performance for each test set.

− indicates that the MSE of TopicVec+LR is too large due to overfitting.

^†^ indicates that *rTopicVec* improved MSE with statistical significance using the Wilcoxn signed-rank test or the Paired-t test at the significance level of 0.1.

^‡^ indicates that *rTopicVec-Ridge* improved MSE with statistical significance using the Paired-t test or the Wilcoxn signed-rank test at the significance level of 0.1.

### Movie rating scores prediction

#### Setup

We additionally evaluated the prediction performance of our proposed models on predicting rating scores from movie reviews by 5-fold cross-validation as performed in sLDA [10]. The dataset was first used in Pang and Lee [19]. The corpus contains 5006 documents. The scores associated with the documents were transformed to approximate normality by taking logs as Blei and McAuliffe [10] did. 10% of the data randomly drawn from the dataset was used as a test set for the open test, and the remaining data was used for cross-validation, which was performed with ten different numbers of topics *K* ∈ {5, 10, 15, 20, 25, 30, 35, 40, 45, 50} to determine the optimal number of topics K* with the smallest average MSE over the five validation tests with rTopicVec. The open test was performed using the model parameters learned with the remaining 90% of the dataset and the optimal number of topics K*. We used MSE as the measure of prediction performance for the validation tests and the open test.

#### Results

**Fig 4:**
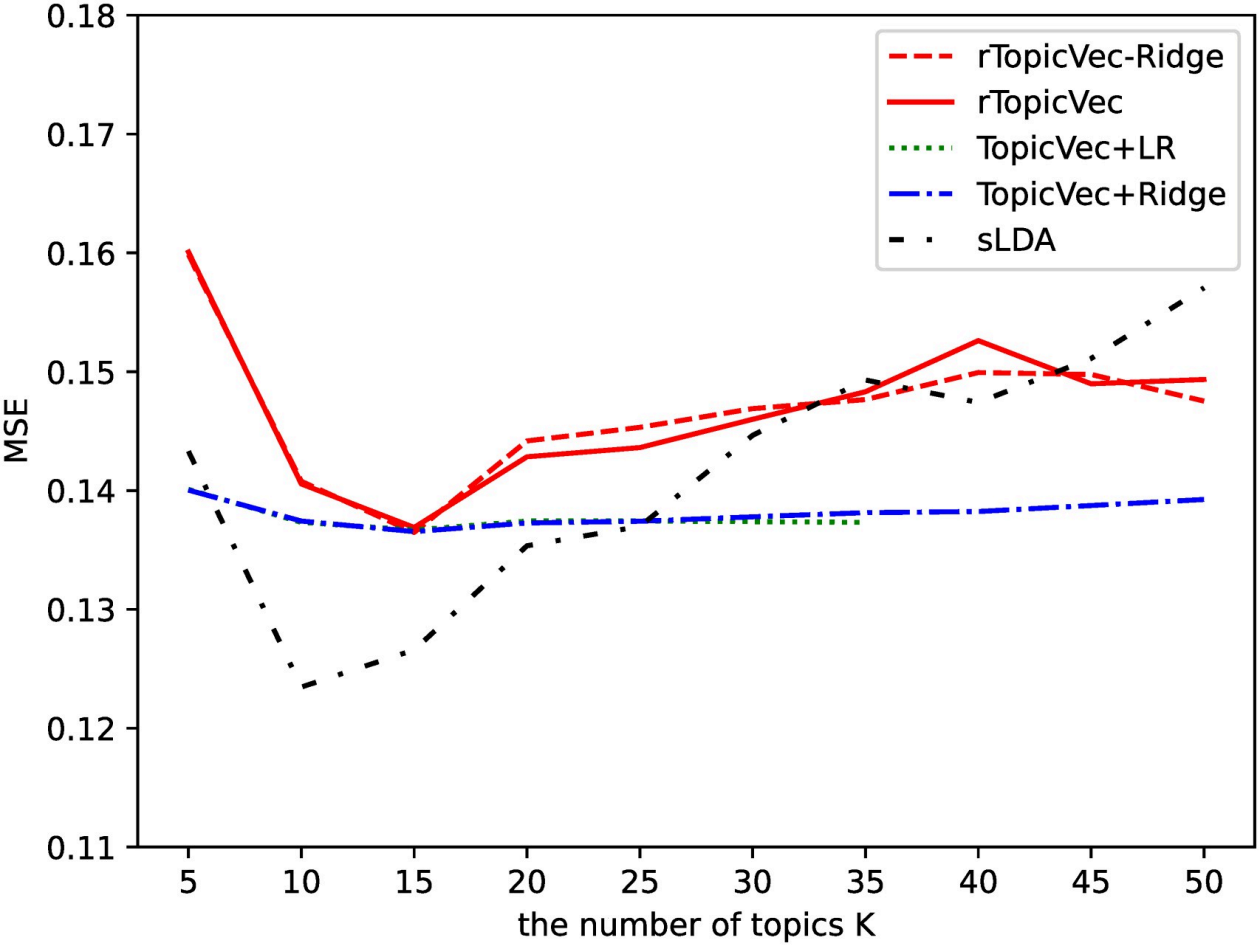
Average MSE for varying number of topics *K* on validation sets of movie rating score prediction.

Fig 4 shows the MSEs obtained as the average of the results of the 5-fold cross-validation. The solid line which denotes rTopicVec shows that *K* = 15 is the optimal number of topics in this prediction problem. The results of TopicVec+LR when *K* > 35 are not shown here since the coefficients are too large due to overfitting. Table 5 presents the topics with two of the highest absolute values of the regression coefficients learned by all the models on the training set when *K* = 15 except TopicVec+LR due to overfitting, and topic coherence measured by NPMI which is commonly applied to verify the topic quality. The NPMI is calculated by using reference counts from an external corpus (English Wikipedia), and is calculated by using the top ten words of each topic, averaging over all the NPMI scores of the topics. Despite the averaged NPMI scores of our proposed models are lower than that of sLDA, the topic coherence increased by incorporating supervisory signals to the unsupervised TopicVec. Table 6 shows that in the open test when *K* = 15, the prediction performance of the proposed model rTopicVec is more accurate than that of the three baselines and two of them with a statistically significant difference. The lowest prediction accuracy achieved by the regularized version rTopicVec-Ridge may be due to underfitting caused by the regularization factor.

**Table 5:** Top 10 words of two topics with highest absolute values of regression coefficients and the topic coherence measured in NPMI on the training set when *K* = 15.

Model (NPMI)	Top 10 words
*rTopicVec* / *rTopicVec-Ridge* (-0.035 / -0.046)	coefficient: 0.307 / 0.303
	review, critical, task, offer, captivating, explanation, movie, director, turn, providing
	coefficient: -0.362 / -0.406
	movie, captivating, task, director, offer, soul, debut, rarely, turn, breakdown
TopicVec+Ridge (-0.052)	coefficient: 0.131
	review, offer, critical, explanation, movie, captivating, director, providing, looked, forces
	coefficient: -0.459
	captivating, task, debut, screwball, trite melodramatic, movie’s, delightfully, perceptive, banal
sLDA (-0.031)	coefficient: 0.524
	captivating, important, director, camera, explanation, task, material, club, twists, movie
	coefficient: -0.329
	opinion, full, important, captivating, cuts, fear, minimum, problem, movie’s, michael

**Table 6:** MSE and sample standard deviation on test set of movie rating score prediction when *K* = 15.

TopicVec+LR	$0.13850_{\pm 0.27308}^{†,‡}$
TopicVec+Ridge	$0.13842_{\pm 0.27389}^{†,‡}$
sLDA	0.13854_±0.29474_
*rTopicVec*	**0.13477**_±0.26940_
*rTopicVec-Ridge*	0.13979_±0.27665_

Bold face indicates the best performance for the test set.

^†^ indicates that the proposed model (*rTopicVec*) improved MSE with statistical significance using the Wilcoxon signed-rank test at the significance level of 0.1.

^‡^ indicates that the proposed model (*rTopicVec*) improved MSE with statistical significance using the Paired-t test at the significance level of 0.1.

## Conclusions and discussions

We proposed rTopicVec, a supervised topic embedding model combining a topic model in the embedding space and linear regression, and furthermore its regularized version rTopicVec-Ridge, to predict the numerical response variables labeled with documents. Through the experiments in predicting stock return rates using news articles and predicting movie ratings using movie reviews, the results showed that the prediction accuracy of our proposed models was higher than that of three baseline models since the topics learned by our proposed models are guided to be predictive of the response variables, and the topics were more coherent than those of TopicVec measured by NPMI and more interpretable to describe a rise/drop in the response variables. In summary, our models are capable of making more accurate predictions on the numerical labels and increasing the interpretability of topics by taking account of the associated labels while reducing the dimensionality of complex text data. We argue that incorporating word embeddings brought our models better prediction accuracy and comparable interpretability than the LDA-based supervised topic model like sLDA, and that learning topics and regression simultaneously brought our models the advantage of higher interpretability and accuracy of the predictions than the models that perform linear regression as post-process. Moreover, the overfitting that occurred in rTopicVec can be alleviated by putting priors on the regression coefficients. We also noticed that there is a huge gap between training and test errors for our proposed models. To narrow this gap as well as to prevent underfitting, the optimal regularization factor needs to be more explored, which will be left for future work. Furthermore, by using the state-of-the-art word embedding based on Transformer [20], the prediction performance and the quality of topics could be improved, which will also be left for future work. Our models can be applied to various applications of predicting response variables using text. Also, our models that assumed regression tasks in this paper can easily be modified to classification tasks.
